# Reduction in massive postpartum haemorrhage and red blood cell transfusion during a national quality improvement project, Obstetric Bleeding Strategy for Wales, OBS Cymru: an observational study

**DOI:** 10.1186/s12884-021-03853-y

**Published:** 2021-05-15

**Authors:** Sarah F. Bell, Rachel E. Collis, Philip Pallmann, Christopher Bailey, Kathryn James, Miriam John, Kevin Kelly, Thomas Kitchen, Cerys Scarr, Adam Watkins, Tracey Edey, Elinore Macgillivray, Kathryn Greaves, Ingrid Volikas, James Tozer, Niladri Sengupta, Iolo Roberts, Claire Francis, Peter W. Collins

**Affiliations:** 1 Department of Anaesthetics, Intensive Care and Pain Medicine, Cardiff and Vale University Health Board, Cardiff, UK; 2 Deputy Director Research Design and Conduct Centre, Centre for Trials Research, College of Biomedical and Life Sciences, Cardiff University, Cardiff, UK; 3 Department of Anaesthetics, Intensive Care and Pain Medicine, Betsi Cadwaladr University Health Board, Glan Clwyd Hospital, Bodelwyddan, UK; 4 Department of Emergency Medicine, Aneurin Bevan University Health Board, Newport, UK; 5 Department of Obstetrics and Gynaecology, Cardiff and Vale University Health Board, Cardiff, UK; 6 Improvement Cymru, Public Health Wales, Cardiff, UK; 7 Department of Midwifery, Singleton Hospital, Swansea, UK; 8 Department of Obstetrics and Gynaecology, Cwm Taf Morgannwg Health Board, Merthyr Tydfil, UK; 9 Department of Anaesthetics and Intensive Care, Aneurin Bevan University Health Board, Newport, UK; 10 Department of Obstetrics and Gynaecology, Betsi Cadwaladr University Health Board, Glan Clwyd Hospital, Bodelwyddan, UK; 11 Institute of Infection and Immunity, School of Medicine, Cardiff University, Cardiff, UK

**Keywords:** Postpartum haemorrhage, Quality improvement, Coagulopathy, Blood transfusion, Viscoelastometry

## Abstract

**Background:**

Postpartum haemorrhage (PPH) is a major cause of maternal morbidity and mortality and its incidence is increasing in many countries despite management guidelines. A national quality improvement programme called the Obstetric Bleeding Strategy for Wales (OBS Cymru) was introduced in all obstetric units in Wales. The aim was to reduce moderate PPH (1000 mL) progressing to massive PPH (> 2500 mL) and the need for red cell transfusion.

**Methods:**

A PPH care bundle was introduced into all 12 obstetric units in Wales included all women giving birth in 2017 and 2018 (*n* = 61,094). The care bundle prompted: universal risk assessment, quantitative measurement of blood loss after all deliveries (as opposed to visual estimation), structured escalation to senior clinicians and point-of-care viscoelastometric-guided early fibrinogen replacement. Data were submitted by each obstetric unit to a national database. Outcome measures were incidence of massive PPH (> 2500 mL) and red cell transfusion. Analysis was performed using linear regression of the all Wales monthly data.

**Results:**

Uptake of the intervention was good: quantitative blood loss measurement and risk assessment increased to 98.1 and 64.5% of all PPH > 1000 mL, whilst ROTEM use for PPH > 1500 mL increased to 68.2%. Massive PPH decreased by 1.10 (95% CI 0.28 to 1.92) per 1000 maternities per year (*P* = 0.011). Fewer women progressed from moderate to massive PPH in the last 6 months, 74/1490 (5.0%), than in the first 6 months, 97/1386 (7.0%), (*P* = 0.021). Units of red cells transfused decreased by 7.4 (95% CI 1.6 to 13.2) per 1000 maternities per year (*P* = 0.015). Red cells were transfused to 350/15204 (2.3%) and 268/15150 (1.8%) (*P* = 0.001) in the first and last 6 months, respectively. There was no increase in the number of women with lowest haemoglobin below 80 g/L during this time period. Infusions of fresh frozen plasma fell and there was no increase in the number of women with haemostatic impairment.

**Conclusions:**

The OBS Cymru care bundle was feasible to implement and associated with progressive, clinically significant improvements in outcomes for PPH across Wales. It is applicable across obstetric units of widely varying size, complexity and staff mixes.

**Supplementary Information:**

The online version contains supplementary material available at 10.1186/s12884-021-03853-y.

## Background

Bleeding after childbirth (postpartum haemorrhage, PPH) is the leading cause of maternal death worldwide [1]. In resource rich countries PPH causes 80% of severe maternal morbidity and its incidence is increasing in many regions [2, 3], including Wales [4], despite international guidance [5–7]. In the UK PPH is described as moderate at 1000 mL blood loss and severe at 2000 mL [6]. Massive PPH is defined as > 2500 mL and, in resource rich countries, is associated with a hysterectomy rate of 6% and intensive care admission in 11.8% of cases [3]. Post-traumatic stress disorder is common after PPH [8]. Multi-professional management of PPH requires the skills of midwives, obstetricians, anaesthetists, healthcare support staff and haematologists working in an effective team. Variations in care are widely reported, with delays in escalation to senior staff a common theme [9, 10]. A recent confidential enquiry identified deficiencies in care, compared to guidelines, in 90% of cases [11].

Postpartum haemorrhage may be exacerbated by haemostatic impairment. A Clauss fibrinogen below 2 g/L is associated with progression of bleeding [12, 13], although clinically significant deficiencies of other clotting factors and platelets are less common [14–16]. In severe PPH, laboratory coagulation results are often too slow to be useful clinically and guidelines recommend the use of empirical treatment with fixed ratios of red blood cells (RBC), fresh frozen plasma (FFP) and platelets, based on data derived from major trauma [5, 6]. This results in many women receiving blood components when haemostasis is normal [17]. Point-of-care haemostasis test results, using viscoelastometry, are available within 10 min and can direct timely and targeted replacement of fibrinogen and avoid unnecessary FFP during PPH [15, 18–20].

International PPH quality improvement projects have been undertaken with the aim of standardising care and improving outcomes. Interventions have included risk assessment, quantitative measurement of blood loss and escalation to senior clinicians, although to date all have used empirical, fixed-ratio transfusion therapy [21–24]. OBS Cymru is a national quality improvement project developed by combining lessons learnt during 10-years of research [17] with themes emerging from international PPH quality improvement projects [21–24]. OBS Cymru introduced an integrated care bundle into all obstetric units in Wales [25]. A key and unique feature was the inclusion of viscoelastometric point-of-care haemostatic tests to guide targeted blood component administration [15, 18–20]. We report the impact of OBS Cymru over a 2-year period.

## Methods

### Intervention

Launched in November 2016, OBS Cymru introduced a PPH care bundle between January and April 2017 into all 12 obstetric units in Wales. These obstetric units support between 500 and 6000 births per year, with about 31,000 births across Wales. The lead research and development office designated OBS Cymru as a quality improvement project and service evaluation according to NHS guidance. Consequently ethical approval and individual consent to collect and report data was not required.

The design, initiation and project interventions have been described in detail previously [25]. OBS Cymru funding provided a Rotem Sigma® point-of-care coagulation device (Instrumentation Laboratories, Werfen, Barcelona, Spain) for use in each obstetric unit. The intervention promoted:

1. Risk assessment of all mothers admitted to delivery suite. The risk assessment tool is available [26].
2. Quantitative measurement of blood loss from delivery using volumetric and gravimetric techniques for all births as opposed to visual estimation. Details of the method and validation data supporting quantitative measurement have been published [4, 25, 27].
3. Escalation of care to senior clinicians, if not already involved, at specified volumes of blood loss. An obstetrician, senior midwife and anaesthetist were required to attend the mother at 1000 mL, at the latest, and a consultant obstetrician and anaesthetist informed at 1500 mL.
4. Point-of-care tests of haemostasis were taken at 1000 mL or earlier for clinical concern. If required, targeted, early replacement of fibrinogen was administered following a published algorithm [26]. Tranexamic acid was infused as soon as abnormal bleeding was recognised and repeated if bleeding continued [28].

The intervention was intended for all births and was not limited to those complicated by abnormal bleeding. It was underpinned by a standardised paperwork proforma that prompted management and created a contemporaneous record of findings and actions [26]. An all Wales guideline reinforced the intervention and standardised obstetric management including uterotonic use [29]. Antenatal anaemia, cell salvage and transfusion policies were unchanged throughout the project.

The Rotem point-of-care coagulation devices were supported by a validated algorithm [15, 17, 18, 26] and were compliant with internal and external quality assurance. A minor revision of the Rotem interpretation algorithm was introduced in 2018 to emphasise the importance of correcting hypofibrinogenaemia before considering FFP [26]. Haemostatic impairment was defined as fibrinogen < 2 g/L, Fibtem A5 < 12 mm or PT or aPTT > 1.5 times normal [6, 30] (equating to PT > 16 or aPTT > 50 s). In autumn 2018 all Rotem devices received a hardware update which was associated with slightly lower Fibtem A5 measurements, the blood product algorthim was not adjusted.

The national team co-ordinated multi-professional training at each unit, as described [25], this training was front-loaded at the start of the project with top-up training throughout the 2 year period. A lead midwife, obstetrician, anaesthetist and haematologist were appointed at each site to support ongoing training and oversee the project locally with the midwifery time funded by the project. The intervention was introduced during the first 6 month period (January to June 2017) and integrated into standard care obstetric units throughout 2017 and 2018. Training covered quantitative measurement of blood loss, escalation of care and interpretation of Rotem results. The OBS Cymru principles were integrated into PROMPT (PRactical Obstetric Multi-Professional Training) for Wales to support sustainability [31]. Annual multi-professional national meetings allowed dissemination of learning and sharing of good practice.

### Data sources

The Welsh Maternity Indicators Dataset (NHS Wales Informatics Service) provided data regarding number of births and mode of delivery. An all Wales OBS Cymru database was established by Improvement Cymru. Women experiencing bleeds ≥1000 mL or in whom there was concern about abnormal bleeding had a limited dataset collected. Women with bleeds ≥1500 mL or who received a transfusion had more detailed information collected (supplementary Figure 1).

In addition, five audits were undertaken to establish the uptake of measured blood loss and use of the paperwork proforma and the risk assessment tool. Audits included up to 30 consecutive women from each obstetric unit, irrespective of blood loss. Individual units were encouraged to advertise the audit and allow the midwife caring for the patient to contribute data following delivery. Audit data was provided by all 12 obstetric units in October 2016 (*n* = 510, before OBS Cymru started), June 2017 (*n* = 455) and June 2018 (*n* = 492), from 11 units in December 2017 (*n* = 405), and from 7 units in December 2018 (*n* = 259).

Anonymous patient surveys were performed in 2017 (June–December) and 2018 (September). Local teams circulated forms to women experiencing PPH ≥1000 mL. Questions explored communication with the mother and her family and areas for improvement in care. A staff questionnaire was circulated to local leadership teams between September and December 2018 to aid understanding of how OBS Cymru had changed local practice.

### Analysis

Data are summarised descriptively with continuous variables reported as median, inter-quartile range (IQR) and range and categorical variables as number and percent or per 1000 maternities. Descriptive data were reported in four 6-month periods between January 2017 and December 2018 to show changes across time. The intervention was being implemented during January to June 2017 and this time period is compared to the last 6 month period, after the intervention had been adopted, in some analyses. This comparison was chosen to reflect project uptake and prior to any analyses being performed. In addition, an interrupted time series analysis was performed to investigate the trend of bleeds ≥2500 mL before and after the intervention was introduced.

Changes in the proportion of women experiencing massive PPH, the number of units of RBC transfused and intensive care admission were analysed by linear regression using the all Wales monthly data. A simple linear relationship was found to be the best model, with higher-order terms not leading to any substantial improvements in the model. The dependent variables were PPH ≥2500 mL, units of RBC transfused in Wales and episodes of intensive care and the independent variable was months in each case. Estimates are reported with 95% confidence intervals (CI). Sensitivity analyses using quasi-Poisson regression models with total maternities as offset provided very similar results. Chi square test was used to compare events in the first and last 6 month periods. Audit results are presented as the percentage of cases where individual interventions were performed with box plots illustrating completion of all components. Funnel plots were used to indicate variations between units. Data were analysed using SPSS version 23, R version 3.6.0 and ggplot2 version 3.2.0.

## Results

### Demographics

Between 1st January 2017 and 31st December 2018, 61,094 women gave birth in Wales. Mode of delivery was unassisted vaginal for 62.6%, instrumental vaginal 9.5%, emergency caesarean section 14.4% and non-emergency caesarean section 13.5%. There were 6024 episodes (98.6/1000 maternities) recorded on the OBS Cymru database because of PPH ≥1000 mL or clinical concern of abnormal bleeding. The number of episodes increased during the project, possibly because of better recognition of total blood loss as quantitative measurement replaced visual estimation (Table 1). For PPH ≥1500 mL, 2209 episodes (36.2/1000 maternities) were reported. The mode of delivery, causes of bleeding and first recorded haemoglobin (Hb) and Clauss fibrinogen remained constant throughout (Table 1).

**Table 1:** Demographics of women experiencing postpartum haemorrhage in Wales and uptake of the OBS Cymru intervention

	Jan-Jun 2017	Jul-Dec 2017	Jan-Jun 2018	Jul-Dec 2018
**Total number of maternities in Wales**	15,204	15,986	14,754	15,150
**Episodes with ≥ 1000 mL blood loss or clinical concern of abnormal bleeding N (/1000 maternities)**	1448 (95.2)	1519 (95.1)	1499 (101.6)	1558 (102.8)
**Episodes with ≥ 1500 mL blood loss N (/1000 maternities)**	547 (36.0)	588 (36.8)	530 (35.9)	544 (35.9)
**Mode of delivery**				
Unassisted vaginal: n (%)	211 (38.6)	228 (38.8)	203 (38.3)	184 (33.8)
Instrumental vaginal: n (%)	115 (21.0)	116 (19.7)	123 (23.2)	147 (27.0)
Non-emergency caesarean section: n (%)	181 (33.1)	194 (33.0)	170 (32.1)	161 (29.6)
Emergency caesarean section: n (%)	40 (7.3)	50 (8.5)	34 (6.4)	51 (9.4)
Not recorded: n (%)	0 (0)	0 (0)	0 (0)	1 (0.2)
**Cause of bleeding for episodes with ≥ 1500 mL blood loss N (%) (NB many bleeds had multiple causes)**				
Uterine atony	323 (59.0)	352 (59.9)	276 (52.1)	315 (57.9)
Surgery related	142 (26.0)	142 (24.1)	135 (25.5)	161 (29.6)
Genital tract trauma	175 (32.0)	185 (31.5)	178 (33.6)	189 (34.7)
Extragenital bleeding only	6 (1.1)	1 (0.2)	12 (2.3)	5 (0.9)
Uterine rupture	4 (0.7)	4 (0.7)	1 (0.2)	0 (0.0)
Placenta praevia	16 (2.9)	8 (1.4)	15 (2.8)	10 (1.8)
Placenta accrete	5 (0.9)	7 (1.2)	5 (0.9)	5 (0.9)
Amniotic fluid embolus	0 (0.0)	1 (0.2)	0 (0.0)	0 (0.0)
Uterine inversion	1 (0.2)	1 (0.2)	0 (0.0)	1 (0.2)
Placental abruption	17 (3.1)	14 (2.4)	17 (3.2)	23 (4.2)
Retained products	63 (11.5)	46 (7.8)	57 (10.8)	45 (8.3)
No cause reported	1 (0.2)	0 (0.0)	1 (0.2)	0 (0.0)
**First blood test after recognition of haemorrhage for PPH ≥ 1500 mL**				
Haemoglobin g/L: Med (IQR), range	104 (93–114) 62–156	104 (94–115) 57–164	103 (93–115) 60–146	102 (92–113) 52–144
Clauss fibrinogen g/L: Med (IQR)	4.2 (3.6–5) 0.3–7.9	4.2 (3.6–4.9) 0.6–7.4	4.3 (3.7–4.9) 0.3–8.3	4.3 (3.7–4.9) 0.4–9.8
**Uptake of OBS Cymru intervention**				
Risk assessment completed. All Wales: n (% of all episodes ≥1000 mL)^a^	23 (1.6)	399 (26.2)	931 (62.1)	1003 (64.4)
Percent completion in individual units: Med (IQR) range ^a^	0.8 (0–1.7) 0–3.3	25 (22–40) 16–59	82 (74–90) 36–96	88 (68–97) 37–99
Paperwork completed. All Wales: n (% of all episodes ≥1000 mL) ^a^	28/1166 (2.4)	503/1274 (39.5)	724/1210 (59.8)	802/1262 (63.5)
Percent completion in individual units: Med (IQR), range ^a^	1.6 (0–3.1), 0–6.1	45.4 (24.0–53.2), 10.2–66.4	53.3 (40.6–79.7) 18.1–97.2	62.4 (45.9–85.1) 10.9–97.6
Blood loss quantitatively measured All Wales: n (% of all episodes ≥1000 mL)	1204 (83)	1409 (93)	1404 (93.7)	1530 (98.2)
Percent blood loss measurement in individual units: Med (IQR) range	76.7 (81.9–90.2) 38–98	93.6 (88.6–97.6) 86–100	93.7 (88.8–98.5) 85–100	99.1 (98.3–99.6) 95–100
Rotem analysis performed: n (% of episodes with bleeds ≥1500 mL)	206 (37.7)	346 (58.8)	380 (71.7)	371 (68.2) ^b^
Percent Rotem analyses performed in individual units: Med (IQR) range	24.9 (16.2–44.9) 10–88	57.2(39.0–74.4) 9–88	59.2 (67.5–83.0) 39–100	85.2 (75.3–90.6) ^b^ 16–96
Rotem analysis requiring intervention and acted on according to algorithm: n (% of episodes with bleeds > 1500 mL)	3/16 (19)	18/35 (51)	19/29 (65)	25/37 (68) ^b^

Legend ^a^:One obstetric unit did not report any data for this intervention and has been excluded from the analysis and ^b^two obstetric units did not return Rotem data between July and December 2018. A more complete dataset was collected for PPH ≥1500 mL and so Rotem analyses and laboratory results are only reported for these cases

### Uptake of OBS Cymru intervention

**Fig. 1:**
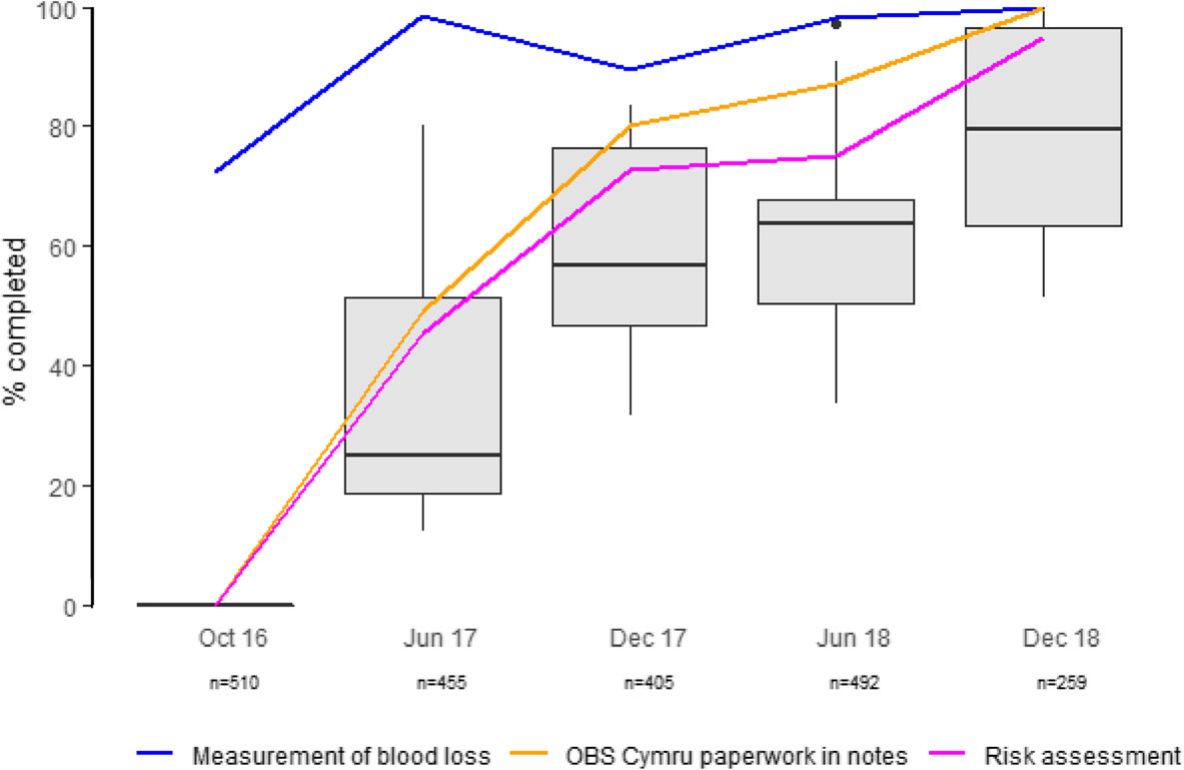
Uptake of OBS Cymru interventions. Legend: The data indicate the percentage of cases where interventions were performed in consecutive women, irrespective of blood loss. Box plots with median, interquartile range and range refer to the combination of paperwork being present in the notes, the risk assessment having been completed and measured blood loss being performed

Uptake increased for all components of the OBS Cymru intervention at all sites. Uptake increased progressively across the 2 year period (Table 1 and Fig. 1). Quantitative blood loss measurement increased from 83 to 98% for PPH ≥1000 mL and Rotem analysis increased from 38 to 68% of episodes with ≥1500 mL blood loss. The proportion of women receiving treatment that was compliant with the blood component algorithm increased from 19 to 68% (Table 1). Audit data of consecutive women, irrespective of blood loss volume, showed the variation between sites in the percentage of maternities where blood loss was measured, risk assessment performed and standardised paperwork used (Fig. 1).

### Severity of postpartum haemorrhage

#### Incidence of massive postpartum haemorrhage

**Fig. 2:**
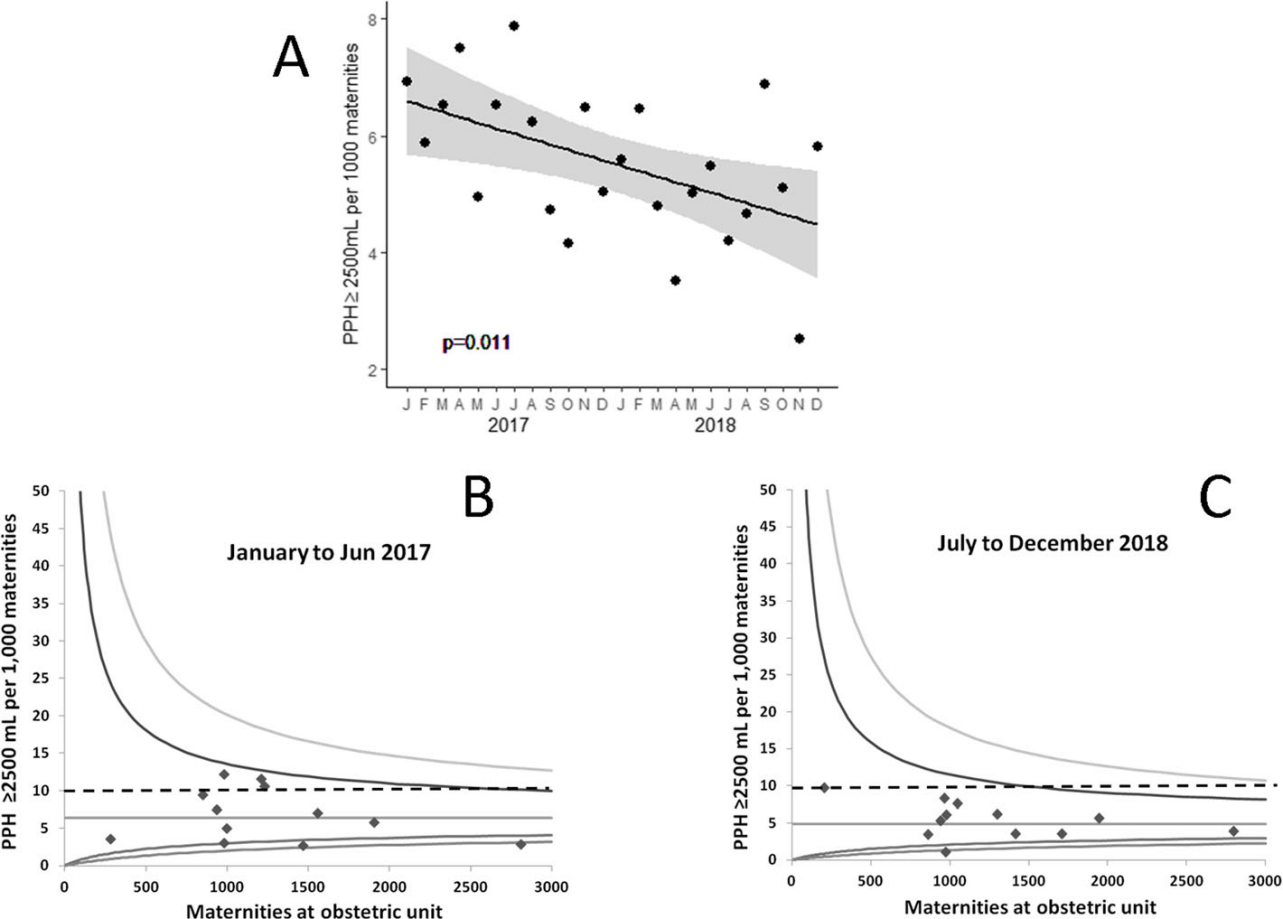
Change in incidence of postpartum haemorrhage > 2500 mL during OBS Cymru. Legend: Panel **a** shows the monthly rates for massive haemorrhage and the fitted regression line with 95% confidence interval shaded in grey. Funnel plots show the incidence of massive postpartum haemorrhages at each obstetric unit in the first (**b**) and last (**c**) 6 month periods of the quality improvement project. The line represents the mean and the limits shown are 2 and 3 standard deviations. The dashed line indicates a massive haemorrhage rate of 10/1000 maternities

Massive PPH (blood loss > 2500 mL) fell from 6.4 to 4.9/1000 maternities between the first and last 6 month periods (Table 2). Regression analysis suggested a progressive fall across the 24 month period with an estimated decrease in massive PPH of 1.10 (95% CI 0.28 to 1.92)/1000 maternities/year (*P* = 0.011) (Fig. 2a). The incidence of massive PPH at each obstetric unit during the first and last 6 months is shown in Fig. 2b and c. These illustrate the overall decrease in massive PPH in Wales and a reduction in the number of obstetric units with massive haemorrhage rates above 10/1000 maternities. More detailed obstetric unit level data are shown in supplementary material Table 1. Information about the incidence of massive PPH in Wales before and after OBS Cymru is shown as an interrupted time series analysis in supplementary Figure 2 demonstrating a statistically significant change in the trend of massive haemorrhage.

**Table 2:** PPH volume, admission to intensive care, hysterectomy and length of stay

	Jan-Jun 2017	Jul-Dec 2017	Jan-Jun 2018	Jul-Dec 2018
PPH ≥1000 mL: n (/1000 maternities)	1386 (91.2)	1480 (92.6)	1412 (95.7)	1490 (98.3)
PPH ≥1500 mL: n (/1000 maternities)	547 (36.0)	588 (36.8)	530 (35.9)	544 (35.9)
PPH ≥2000 mL: n (/1000 maternities)	228 (15.0)	232 (14.5)	228 (15.5)	209 (13.8)
PPH ≥2500 mL: n (/1000 maternities)	97 (6.4)	92 (5.8)	76 (5.2)	74 (4.9)
Admission to intensive care for PPH: n (/1000 maternities)	10 (0.66)	12 (0.75)	9 (0.61)	6 (0.40)
Hours in intensive care for PPH: n (/1000 maternities)	322.3 (21.2)^a^	290 (18.1)	180 (12.2)	124 (8.2)
Hysterectomy associated with PPH: n (/1000 maternities)	5 (0.33)	3 (0.19)	8 (0.54)	3 (0.20)
Length of hospital stay (days) for women with PPH ≥1500 mL: Med (IQR), range	2.09 (1.37–3.27) 0.08–13.3	2.01 (1.29–3.22) 0.02–13.3	2.12 (1.45–3.23) 0.12–26.6	2.11 (1.44–3.20) 0.05–28.9

Legend: The number of PPHs (/1000 maternities) between 1000 and 1499 mL was 55.2, 55.7, 59.8 and 62.4 in each six-month period. ^a^ one woman spent 168 h on intensive care between January and June 2017

The all Wales incidence of PPH ≥1000 mL increased, PPH ≥1500 mL remained stable and ≥ 2000 mL fell slightly (Table 2) throughout the 2 year period. The ratio of moderate PPH (1000 mL) vs massive PPH (> 2500 mL) fell throughout the 2 year period; in the first 6 months, 97/1386 (7.0%), second 6 months 92/1480 (6.2%), third 6 months 76/1412 (5.3%) and last 6 months, 74/1490 (5.0%) (*P* = 0.021 comparing first and last 6 months).

#### Intensive care admission, hysterectomy and length of hospital stay

The estimated decrease in intensive care admission was 0.31 (95% CI: − 0.21–0.84)/1000 maternities/year (*P* = 0.23). The total time spent in intensive care fell but this was due mainly to a prolonged stay of 168 h for one woman in the first 6 months (Table 2). There were 22 hysterectomies in total (0.36/1000 maternities), of which 19 were associated with PPH (0.31/1000) (Table 2). Of these hysterectomies, 11/19 (58%) were for placenta accreta, increta or praevia and are likely to represent appropriate care [6, 7] The eight hysterectomies for PPH that were unrelated to abnormal placentation occurred at 0.13, 0.06, 0.27 and 0.07/1000 maternities in each 6 month period. These numbers are too small for meaningful comparison. The length of hospital stay for women with PPH ≥1500 mL was similar throughout (Table 2).

#### Transfusion of red blood cells and blood components

**Fig. 3:**
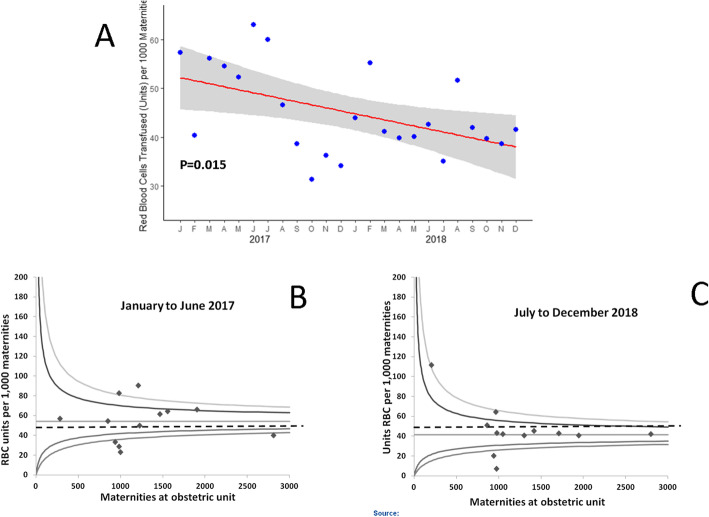
Change in incidence of red blood cell transfusion during OBS Cymru. Legend: Panel **a** shows the monthly rates for red blood cell transfusion and the fitted regression line with 95% confidence interval shaded in grey. Funnel plots show the incidence of RBC transfusion for postpartum haemorrhages at each obstetric unit in the first (**b**) and last (**c**) 6 month periods of the quality improvement project. The line represents the mean and the limits shown are 2 and 3 standard deviations. The dashed line represents a RBC transfusion rate of 50 units/1000 maternities

The proportion of women transfused RBCs for PPH fell from 350/15204 (2.3%) to 268/15150 (1.8%) between the first and last 6 month periods, (*P* = 0.0010). The total number of units of RBCs transfused in Wales fell from 54.1 to 40.2/1000 (Table 3). Regression analysis estimated that the number of units of RBCs transfused for PPH fell by 7.4 (95% CI 1.6–13.2)/1000 maternities/year, *P* = 0.015 (Fig. 3a and Table 3). The total number of units of RBC/1000 maternities transfused at each obstetric unit in the first and last 6 months of the project is shown in Fig. 3b and c. The number of obstetric units that transfused ≥50 units of RBC per 1000 maternities fell from 8/12 to 3/12 (*P* = 0.041). Despite the reduction in RBC transfusions, the proportion of women with a lowest Hb < 80 g/L did not increase (Table 3).

**Table 3:** Transfusion and haematological results for postpartum haemorrhage

	Jan-Jun 2017	Jul-Dec 2017	Jan-Jun 2018	Jul-Dec 2018
**Transfusion of red blood cells and blood components**				
Number of women transfused red blood cells: n (/1000 maternities)	350 (23.0)	270 (16.9)	278 (18.8)	268 (17.7)
Total number of units of red blood cells transfused: n (/1000 maternities)	823 (54.1)	656 (41.0)	636 (43.1)	609 (40.2)
Number of women transfused ≥5 units red blood cells: n (/1000 maternities)	16 (1.1)	14 (0.9)	14 (1.0)	11 (0.7)
Number of women transfused FFP: n (/1000 maternities)	26 (1.7)	20 (1.3)	21 (1.4)	15 (1.0)
Total number of units of FFP transfused: n (/1000 maternities)	87 (5.7)	78 (4.9)	74 (5.0)	37 (2.4)
Number of women transfused fibrinogen: n (/1000 maternities)	22 (1.5)	19 (1.2)	17 (1.2)	30 (2.0)
Total number of grams of fibrinogen transfused: n (/1000 maternities)	94 (6.2)	103 (6.4)	89 (6.0)	137 (9.0)
Number of women transfused cryoprecipitate: n (/1000 maternities)	6 (0.4)	3 (0.2)	2 (0.1)	5 (0.3)
Total number of units of cryoprecipitate transfused: n (/1000 maternities)	14 (0.9)	8 (0.5)	4 (0.3)	9 (0.6)
Number of women transfused platelets: n (/1000 maternities)	12 (0.79)	8 (0.50)	6 (0.41)	7 (0.46)
Total number of units of platelets transfused: n (/1000 maternities)	20 (1.3)	13 (0.8)	10 (0.7)	9 (0.6)
**Haematological results**				
Lowest Clauss fibrinogen results: Med (IQR), range	4.2 (3.4–5), 0.3–9.2	4.1 (3.4–4.8), 0.5–8.1	4.2 (3.5–4.7), 0.3–8	4.2 (3.5–4.8), 0.3–9.8
Number with lowest fibrinogen ≤2 g/L n/reported results (% of reported results)	24/383 (6.3)	18/399 (4.5)	17/435 (3.9)	22/459 (4.8)
Longest PT results: Med (IQR), range	10.7 (10.3–11.3), 9.2–22.4	10.6 (10.3–11.1), 9.1–80	10.4 (10.1–10.9), 8.3–18.4	10.4 (10–10.9), 9–19.5
Number with longest PT > 16 s n/reported results (% of reported results)	5/384 (1.3)	5/388 (1.3)	5/434 (1.2)	4/458 (0.9)
Longest aPTT results: Med (IQR), range	25.9 (24.1–27.6), 20–84	25.6 (23.9–27.5), 19.5–143	25.1 (23.7–27.2), 19.3–105	24.9 (23.5–26.8), 18.7–42.5
Number with longest aPPT > 50 s n/reported results (% of reported results)	4/384 (1.0)	3/388 (0.8)	2/435 (0.5)	0/458 (0.0)
Lowest Fibtem A5 results: Med (IQR), range	21 (18–25), 4–49	21 (17–24), 2–63	22 (19–25), 2–55	18 (16–21), 0–60
Number with lowest Fibtem A5 < 12 mm n/reported results (% of reported results)	14/205 (6.8)	26/344 (7.6)	23/378 (6.1)	30/369 (8.1)
Longest Extem CT results: Med (IQR), range	57 (52–62), 17–120	57 (52–63), 38–147	56 (52–61), 30–300	55 (51–61), 11–481
Number with longest Extem CT > 75 s n/reported results (% of reported results)	8/205 (3.9)	15/344 (4.4)	19/378 (5.0)	9/369 (2.4)
Lowest haemoglobin results: Med (IQR), range	91(71–100), 46–135	89 (80–100), 54–139	88 (78–98), 47–137	88 (79–100), 52–137
Number with lowest haemoglobin < 80 g/L n/reported results (% of reported results)	55/203 (27.1)	99/327 (30.3)	82/353 (23.2)	55/306 (18.0)

The proportion of women receiving FFP fell by 42% (*P* = 0.088) and the use of fibrinogen concentrate increased by 37% (*P* = 0.26) between the first and last 6 months (Table 3). The decrease in FFP usage occurred mainly in the final 6 months after clinicians had been encouraged to correct fibrinogen before infusing FFP [26]. Infusion of cryoprecipitate and platelets was very uncommon (Table 3).

#### Haemostatic impairment

The number of laboratory coagulation tests reported increased over time and so differences between 6 month periods must be interpreted with caution. Despite this, the restrictive use of FFP was not associated with an increase in haemostatic impairment as demonstrated by the lowest fibrinogen and the longest PT/aPTT (Table 3). The median Fibtem A5 was 3 mm lower and more women had a Fibtem A5 < 12 mm in the last 6 months, this is discrepant to the laboratory fibrinogen results and probably reflects the change in Fibtem A5 after the Rotem devices were updated. This may have affected the number of women receiving fibrinogen concentrate which increased in the last 6 months (Table 3).

#### Maternal and staff feedback

Results of the patient survey undertaken in 2017 have been reported [25]. Eight obstetric units collected 47 patient surveys in September 2018, mean (range) blood loss 1716 (1029–5743) mL (data was unavailable regarding the survey response rate). In total 66% of women remembered being told that they were having abnormal bleeding and although 95% (39/41) felt well supported during the PPH, 29% (8/28) said that care could have been improved and better communication with mother and partner was suggested. No mother reported that the process of measuring blood loss or the escalating the multi-professional team response to her bleeding had a negative impact on the birth experience.

Local leadership staff survey, response rate 29/46 (63%), reported that OBS Cymru had changed individual and unit level management of PPH. Table 4 shows the components of the intervention that clinicians thought had led to change.

**Table 4:** Contribution of intervention to practice change during OBS Cymru

Intervention	Importance to practice change, *n* = 29 (1- not important, 5- most important) Median (IQR)
Quantitative measurement of blood loss	5 (4–5)
Team working	5 (3–5)
Point-of-care testing of coagulation	5 (3–5)
Paperwork proforma	4 (1–4)

Legend: Responses were 37.9% midwifery, 37.9% anaesthesia, 17.2% obstetrics, 6.9% haematology. Free text responses describing changes in individual practice included ‘awareness of ongoing blood loss’, ‘proactive rather than reactive’, ‘consistent management’, ‘appropriate product administration’, ‘communication and team-working’. Barriers to implementation were reported by 69% (20/29) with the most common theme being training (70%, 14/20). This was also the leading response for overcoming barriers 53.5% (8/15)

## Discussion

OBS Cymru was a national quality improvement project that aimed to reduce morbidity associated with PPH by introducing a care bundle into all 12 obstetric units in Wales. There were clinically and statistically significant reductions in massive haemorrhage across Wales with a 29% fall in the number of women progressing from moderate to massive PPH. The number of women exposed to RBC transfusion fell by 22% and the number of units of RBC transfused for PPH decreased by 26%.

Adoption of the whole care bundle progressively improved throughout the project with quantitative blood loss measurement approaching 100% during the last 6 months. This technique is more accurate than visual estimation, which tends to under-report actual volume, especially for large bleeds [32–37]. The increase in bleeds ≥1000 mL is likely to be a consequence of relative under-reporting during the early stages of OBS Cymru when quantitative measurement was being introduced. Similarly, recognition of massive haemorrhage is likely to have improved over time and so the reduction in the rate of PPHs ≥2500 mL may be an under-estimate.

The main strength of this report is that it represents service change implemented across an unselected real-world national cohort of women. It includes all women giving birth in Wales and all obstetric units irrespective of size, case mix and staffing levels. Women who gave birth in the community and experienced bleeding were transferred to an obstetric unit and are included in the results. The improvements in outcomes are internally consistent and continued throughout the project. The population, mode of delivery and cause of bleeding are similar to many high resource countries making the results widely applicable.

This report describes the changes observed over the course of the project and it cannot be known for certain that the improved outcomes were the result of the care bundle. However, improvements of the size observed are very unlikely to have happened simultaneously in multiple centres by chance and progressive improvement in outcomes coincided with the progressive adoption of the intervention. The largest improvements in massive haemorrhage occurred in obstetric units with high initial rates possibly, in part, due to regression towards the mean.

The blood component algorithm emphasised early treatment of hypofibrinogenaemia in line with previous studies and guidelines [5, 6, 12, 15, 18, 20, 38]. In the last 6 month period the Rotem devices had a hardware update associated with a fall in median Fibtem. Clinicians should be wary of variations between and within point-of-care devices and engage local laboratory expertise and monitor local normal ranges [19].

Quantitative measurement of blood loss alone does not improve outcomes [39]. However, when integrated into a care bundle such as OBS Cymru, real time accurate knowledge of blood loss acts as an enabler to prompt teams to escalate care according to guidelines [6, 38]. The changes in massive haemorrhage and concurrent uptake of OBS Cymru interventions suggest that measuring blood loss and using Rotem, facilitated by multi-professional team attendance at the bedside, were important factors. This was supported by feedback from clinicians, with blood loss measurement, team working and point-of-care coagulation tests stated to be the most influential changes to practice.

The rate of hysterectomy for PPH remained low throughout the project (0.31/1000 maternities). Consideration of early hysterectomy in cases of abnormal placentation is advocated by guidelines and 58% of hysterectomies were reported to have abnormal placental implantation [6, 7]. Other studies report a hysterectomy rate of 0.6–1/1000 maternities [3, 21] demonstrating that hysterectomies were uncommon in Wales before OBS Cymru and this may explain why improvements were not seen.

The number of women transfused RBCs for PPH fell by 22%, equivalent to about 160 women in Wales avoiding transfusion annually. RBC transfusion fell to 40 units/1000 maternities compared to a UK average of about 100 units/1000 maternities. Despite this, the lowest Hb during a PPH was similar throughout the 2 year period and the proportion of women with Hb below 80 g/L did not increase, suggesting the reduction in transfusion reflected reduced bleeding rather than withholding RBCs inappropriately. Treatment of antenatal anaemia was consistent throughout the project supported by the finding that the first recorded Hb remained unchanged across the 2 year period.

RBCs, FFP and platelets are often transfused in fixed-ratios for major PPH [5, 6, 21, 24, 40–43] based on data derived from major trauma [44, 45] PPH differs from major trauma because at term women have an expanded circulating blood volume and are hypercoagulable [46, 47] and can maintain adequate haemostasis despite moderate blood loss [15, 16]. Clinically significant deficiency of coagulation factors other than fibrinogen is uncommon in PPH and fixed-ratio transfusion algorithms may result in women receiving FFP with normal coagulation [17]. In OBS Cymru PT/aPTT > 1.5 times normal was seen in 1% of women experiencing a PPH ≥1500 mL whilst fibrinogen < 2 g/L occurred in about 5%, consistent with other studies [13, 15, 16] In OBS Cymru Extem clot time was used to guide FFP infusion, with a 42% reduction in women receiving FFP. This occurred mainly in the last 6 months after the importance of correcting hypofibrinogenaemia before FFP administration was emphasised. During this time fewer women had PT/aPTT > 1.5 times normal demonstrating that conservative use of FFP during PPH, guided by point-of-care tests, does not increase haemostatic impairment [15].

Other large-scale quality improvement projects for PPH have combined risk assessment, measured blood loss, standardised escalation and empirical, as opposed to targeted, blood component resuscitation [21, 22, 24]. These initiatives have shown that, with high adoption of the interventions, severe morbidity can be reduced [21]. Sites adopting The California Maternal Quality Care Collaborative care bundle reported a 21% reduction in severe maternal morbidity [24]. The Association of Women’s Health, Obstetric, and Neonatal Nurses PPH Project implemented a care bundle into 58 hospitals over 18 months. There was variable uptake and no statistically significant difference in maternal morbidity [22]. The lack of dedicated multi-professional time was identified as a barrier. The use of multi-professional leadership teams in OBS Cymru, embedded at both a local and national level, with dedicated time to lead change, contributed to the speed, uptake and success of the project.

The integrated care bundle introduced universal risk assessment to identify mothers of increased risk of PPH, whilst quantitative measurement of blood loss enabled early recognition of abnormal bleeding and progression. This facilitated escalation to more experienced midwives and obstetricians to treat the underlying cause of bleeding earlier whilst anaesthetists focused on timely resuscitation. Point-of-care tests identified cases of hypofibrinogenaemia, allowing targeted and rapid correction of coagulopathy whilst avoiding inappropriate FFP in the majority. Early identification of normal coagulation facilitated escalation of obstetric measures to control bleeding.

## Conclusions

A care bundle for the management of PPH, that included point-of-care tests of coagulation to guide the treatment of coagulopathy, was introduced as a national quality improvement project involving more than 30,000 maternities annually. Clinically significant improvements in PPH outcomes, including rates of massive haemorrhage and RBC transfusion are achievable on a national level using quality improvement methodology. Obstetric units of all size and case mix implemented and benefitted from the care bundle with improved national outcomes. These results suggest that trends towards increasing incidence of severe PPH seen over recent years can be reversed by structured multi-professional team interventions. A cluster randomised trial is needed to investigate whether the OBS Cymru care bundle improves outcomes for PPH compared to standard care.

## Supplementary Information

**Additional file 1: Figure S1.** Data collection proforma for postpartum haemorrhage of 1000 mL and 1500 mL. **Figure S2.** Interrupted time series analysis of massive postpartum haemorrhage (> 2500 mL) before and after OBS Cymru.

## Data Availability

The datasets generated and/or analysed during the current study are not publicly available because there are held on a quality improvement database hosted by Cardiff and Vale University Health Board. Anonymised data are available from the corresponding author on reasonable request.

## Abbreviations

PPH: Postpartum haemorrhage
RBC: red blood cells
FFP: fresh frozen plasma
