# Supplementary material for: Reduction in massive postpartum haemorrhage and red blood cell transfusion during a national quality improvement project, Obstetric Bleeding Strategy for Wales, OBS Cymru: an observational study
Source: BMC Pregnancy Childbirth. 2021 May 15;21:377. doi: 10.1186/s12884-021-03853-y (PMC8126150; doi:10.1186/s12884-021-03853-y)
Supplement: Supplementary file 1 — Additional file 1: Figure S1. Data collection proforma for postpartum haemorrhage of 1000 mL and 1500 mL. Figure S2. Interrupted time series analysis of massive postpartum haemorrhage (> 2500 mL) before and after OBS Cymru. [file 12884_2021_3853_MOESM1_ESM.docx]

**Supplementary material**

Figure 1. Data collection proforma for postpartum haemorrhage of 1000 mL and 1500 mL

*Change in blood loss of 2500 mL of more at individual sites*

Four obstetric units had a massive PPH (>2500mL) rate below 4 per 1000 maternities in the first month of 2017, all of whom observed an increase in the rate in the last 6 months of 2018. In 2 of these sites the rate remained below 4 per 1000 maternities, whilst 1 unit saw an increase to 6.0 per 1000 maternities. The smallest obstetric unit in Wales reported a substantial increase in PPH rate, this was due to two events in the final 6 months compared to one event in the first 6 months and so may not be representative. This unit also reported very low use of ROTEM in cases of PPH > 1.5L PPH in comparison to the other sites. The 8 obstetric units with rates above 5 per 1000 maternities in the first 6 months of 2017 observed a reduction in massive PPH rate. In these obstetric units, completion of the paperwork in cases of PPH was the least adopted intervention, whilst risk assessment, measurement of blood loss and use of ROTEM were widely adopted.

Table 1.

| Size of obstetric unit (no. maternities July-Dec 2018) | >2500 mL PPH rate per 1000 maternities Jan- June 2017 | Change in >2500 mL PPH rate per 1000 maternities rate between Jan- June 2017 to July-Dec 2018 | % of women who had a risk assessment | % of women who had the paperwork completed | % of women who had quantitative measurement of blood loss | % of women who experienced a PPH >1500mL and had a ROTEM test performed |
| --- | --- | --- | --- | --- | --- | --- |
| 1418 | 2.6 | +1.0 | 94.4 | 66.4 | 100 | 84.2 |
| 2799 | 2.9 | +1.0 | na | Na | 97.6 | 86.2 |
| 978 | 3.0 | +3.0 | 97.3 | 96.4 | 99.1 | 95.7 |
| 206 | 3.5 | +6.2 | 68.0 | 52.0 | 100 | 18.2 |
| 972 | 5.3 | -4.2 | 97.0 | 95.5 | 98.5 | na |
| 1947 | 6.0 | -0.2 | 97.6 | 97.6 | 99.0 | na |
| 939 | 7.2 | -1.8 | 63.4 | 42.7 | 100 | 96.9 |
| 1710 | 7.4 | -3.8 | 60.0 | 32.8 | 99.5 | 69.7 |
| 864 | 9.2 | -6.2 | 88.3 | 53.2 | 97.4 | 88.9 |
| 1302 | 10.7 | -4.4 | 86.2 | 74.8 | 98.7 | 81.1 |
| 1048 | 11.6 | -4.0 | 68.4 | 62.4 | 97.4 | 73.3 |
| 967 | 12.0 | -3.7 | 99.1 | 10.1 | 99.1 | 91.2 |

Legend. Table illustrating the individual obstetric unit level change in massive PPH rate and the uptake of the OBS Cymru complex intervention. NA is not available. Data source: OBS Cymru database

Figure 2 Interrupted time series analysis of massive postpartum haemorrhage (>2500 mL) before and after OBS Cymru

**OBS Cymru**


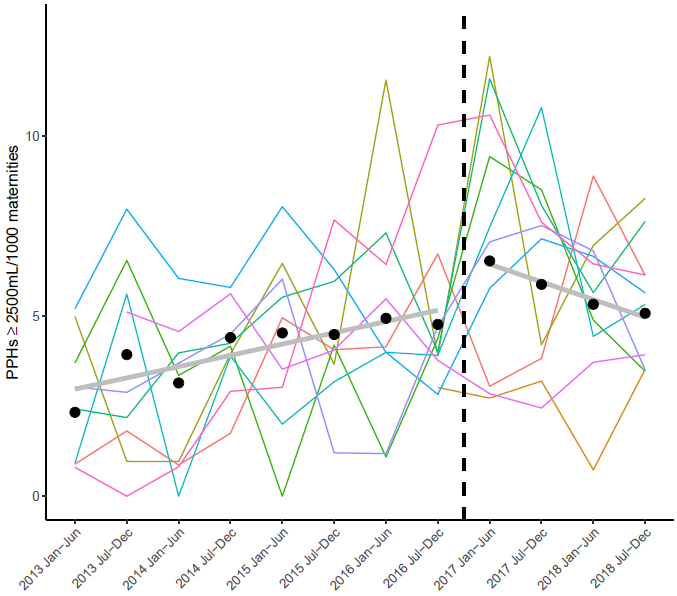


Legend:The data between 2013 and 2016 were obtained retrospectively from obstetric units by the OBS Cymru team. There was insufficient data from two obstetric units and these were not included. Coloured lines are for individual obstetric units and the grey line is a segmented linear mixed-effects model fitted to aggregate unit-level data with random effects for obstetric units (thus weighted by number of maternities per unit at each time point).

The increase in incidence prior to the start of OBS Cymru (p=0.005) is likely to be due to the introduction of quantitative measurement of blood loss leading to improved recognition of massive haemorrhage. The retrospective data from the obstetric units and the prospective data from the OBS Cymru database cannot be directly compared because they have been collected using different methods. The comparison, however, supports the conclusion that there was a change in the trend of massive haemorrhage associated with the introduction of OBS Cymru (p=0.004).
